# Understanding and guiding technology use in dementia: a pan-European mapping and consensus study

**DOI:** 10.3389/frdem.2025.1735879

**Published:** 2025-12-17

**Authors:** Canaan Tsabary, Duygu Sezgin, Anthea Innes, Dianne Gove, Ana Diaz, Lia Fernandes, Ana Barbosa, Michael P. Craven, Horst Christian Vollmar, Laila Øksnebjerg, Louise Hopper

**Affiliations:** 1 School of Psychology, Dublin City University, Dublin, Ireland; 2 School of Nursing and Midwifery, University of Galway, Galway, Ireland; 3 Centre for Rural Health Science, University of the Highlands and Islands, Inverness, United Kingdom; 4 Gilbrea Centre for Studies in Aging, McMaster University, Hamilton, ON, Canada; 5 Alzheimer Europe, Senningerberg, Luxembourg; 6 Department of Clinical Neurosciences and Mental Health, Faculty of Medicine, RISE-Health, University of Porto, Porto, Portugal; 7 Psychiatry Service, São João University Hospital, Porto, Portugal; 8 Centre for Applied Dementia Studies, University of Bradford, Bradford, United Kingdom; 9 NIHR MindTech HRC, Institute of Mental Health, University of Nottingham, Nottingham, United Kingdom; 10 Human Factors Research Group, Faculty of Engineering, University of Nottingham, Nottingham, United Kingdom; 11 Institute of General Practice and Family Medicine (AM RUB), Ruhr University Bochum, Bochum, Germany; 12 Department of Psychology, University of Copenhagen, Copenhagen, Denmark

**Keywords:** assistive technology, dementia, COVID-19, mapping exercise, Delphi method, expert consensus, public involvement

## Abstract

**Introduction:**

Dementia is a leading cause of disability worldwide, and its prevalence is expected to rise significantly by the year 2050. Assistive technologies (AT) have emerged as promising tools to promote independence and quality of life. The COVID-19 pandemic prompted an increased uptake of AT among people with dementia, exposing important limitations in digital literacy, accessibility, and support.

**Methods:**

This pan-European study mapped recent research initiatives involving digital technology use by people with dementia during the pandemic and synthesised a set of recommendations for supporting the use of AT by people with dementia, and its development, using the Delphi method.

**Results:**

The mapping exercise identified 28 relevant projects, highlighting the types of technologies used during the pandemic and the settings in which they were implemented. Video-conferencing platforms were the most reported projects. More than half of the projects and initiatives (*n* = 17) were adapted to include digital technologies due to the pandemic. The subsequent Delphi consensus study incorporated input from experts by experience and produced 18 evidence-based recommendations, adapted from this mapping exercise and a previous scoping review.

**Discussion:**

Key findings emphasise involving people with dementia in technology design, ensuring equitable access, and providing adequate training and support. The recommendations offer a practical, consensus-based framework to improve the efficacy of AT adoption, with implications extending beyond pandemic contexts to improve dementia care globally.

## Background

Dementia is one of the leading causes of disability worldwide (Wimo et al., 2023) and, according to the World Health Organization (2017) and World Alzheimer Report 2023 (Long et al., 2023), its global prevalence is expected to rise to 139 million by the year 2050. As such, there has been a growing emphasis on enhancing the independence of people with dementia in order to improve their quality of life and mitigate the mounting burden on healthcare systems. The use of assistive technologies (AT) has emerged as a promising approach to promote well-being in this context. The category of AT refers to “any item, piece of equipment, product or system driven by electronics, whether acquired commercially, off-the-shelf, modified or customised, that is used to help people living with dementia in dealing with the consequences of dementia” (Meiland et al., 2017; Neal et al., 2025). The adoption of AT can help to support individuals’ freedom and safety living in their own homes (Dada et al., 2022; Löbe and AboJabel, 2022) – by, for example, introducing memory aids (e.g., audible medication reminders); orientation aids (e.g., electronic calendar clocks); safety devices (e.g., motion-sensor night lights), and more (Assistive Technology and Dementia, 2023).

Several important barriers to well-being among people with dementia—such as memory deficits, reduced mobility, and social isolation (Rai et al., 2022)—were significantly exacerbated during the COVID-19 pandemic and the public health restrictions imposed therein (Curelaru et al., 2021). During this time, there was an increase in technology uptake within this population (Barbosa et al., 2023; Gedde et al., 2021), particularly in digital technologies (Chirico et al., 2022). For example, Kalicki et al. (2021) found that over one-third of community-dwelling people with dementia engaged in video-based telehealth appointments for the first time during the pandemic. Research suggests that the use of AT elicits important benefits for people affected by dementia (PABD; i.e., people with dementia and their carers/supporters) both within and beyond a health pandemic (Barbosa et al., 2023; Pappadà et al., 2021); however, salient limitations in the use and development of AT were highlighted by the emergence of COVID-19. The accelerated uptake of technology among people with dementia during the pandemic underscored the critical need for accessible, well-designed technologies, as many individuals lacked adequate digital literacy and support for this use (Barbosa et al., 2023). This points to the value of involving people with dementia in the design and implementation of AT, which has been emphasised in the literature (Meiland et al., 2017; Neal et al., 2025; Span et al., 2013). Despite this, salient gaps remain in understanding how technologies were used and people with dementia during the pandemic. Therefore, there is a need to explore which technologies were implemented and develop recommendations for their design, implementation, and support. This is an important way to support the well-being of people with dementia at all stages of their disease, while addressing their changing needs.

A task force on AT was established as part of INTERDEM, which is an interdisciplinary European research network of more than 200 members, collaborating to develop and carry out pan-European research on early, timely, and quality psychosocial interventions in dementia (INTERDEM, 2024). A COVID-19 subgroup of the AT task force was set up to conduct a project (UTeC19) exploring the projects and initiatives related to the uptake of digital technology among people with dementia in Europe during the COVID-19 pandemic. This subgroup conducted a scoping review to identify the literature on studies undertaken since the onset of the COVID-19 pandemic in order to identify and collate variables to include types of technology used by individuals with dementia, methodologies, setting, and participant characteristics (Barbosa et al., 2023). This current study reports the further work of this subgroup – a mapping exercise – conducted to identify projects, including recently concluded or ongoing studies and initiatives, that were reported by the INTERDEM members and their professional networks during the pandemic to add to the published evidence identified in the scoping review. Here, we also present the synthesis of a set of recommendations for developing and supporting the use of AT by people with dementia. These statements were developed through a Delphi study, which incorporates evidence gathered from the scoping review and mapping exercise, with additional support from a patient public expert panel. This consensus approach allows for a more robust and widely accepted set of recommendations that are supported empirically, through multiple rounds of feedback, and grounded in the lived experiences of people affected by, and working with dementia. The current paper, therefore, serves to collate the research investigating the role of AT in the lives of PABD during the COVID-19 pandemic, as well as providing a set of actionable recommendations on the use of and approach to AT in dementia care. The current report adheres to the ACCORD (ACcurate COnsensus Reporting Document) guideline for reporting consensus methods (Gattrell et al., 2024).

## Aims

The aims of this study were to (a) map and characterise any projects (i.e., recently concluded or ongoing studies and initiatives conducted by members of INTERDEM and their professional networks) involving people with dementia during the COVID-19 pandemic which incorporated the use of technology. Furthermore, we aimed to (b) synthesise a set of evidence-based recommendations, validated through a Delphi consensus process, for the development and use of AT by PABD.

## Method

**Figure 1:**
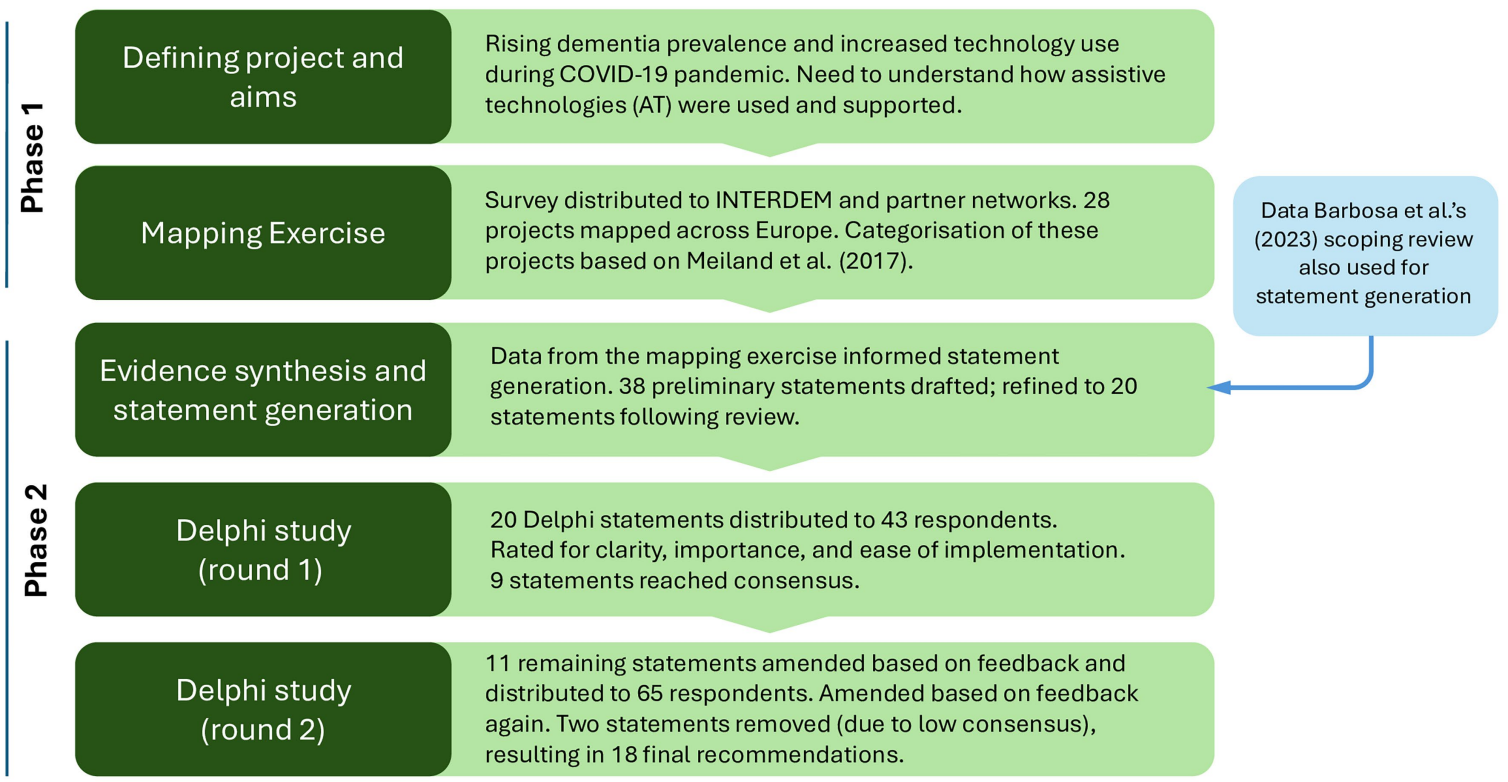
Flowchart of project methods.

This study consisted of a mapping exercise, followed by a Delphi study. The mapping exercise provided insights into projects and initiatives involving technology use by people with dementia during the pandemic, which, together with evidence from Barbosa et al.’s (2023) scoping review, formed the basis of the content for the Delphi statements for the preparation of the recommendations, validated in the Delphi consensus phase. This integration ensured that the recommendations were grounded in current practice, informed by research. See Figure 1 for an outline of the research process.

### Phase 1: mapping exercise

The COVID-19 subgroup of the Assistive Technologies (AT) task force conducted a survey circulated to the wider INTERDEM group (i.e., not limited to the AT task force) in order to identify and map recently completed and/or ongoing research studies, projects and initiatives that incorporate the use of technology by people with dementia to explore the uptake of technology among people with dementia during the COVID-19 pandemic in Europe.

#### Participants

Members of INTERDEM, who are researchers actively engaged in dementia research in Europe and beyond, were invited to take part in the survey. They were also encouraged to share the survey in their professional networks to increase participation and representation of research studies.

#### Data collection

An online surveying platform (QuestionPro, licensed to the University of Galway) was used. The data was collected between 21 December 2021 and 14 March 2022. An invitation to participate in the survey was sent by email to over 200 members of INTERDEM by the INTERDEM secretary. Monthly reminders were sent to increase the response rate. In addition, the survey was promoted online via the INTERDEM social media account. Members of INTERDEM were encouraged to further share the survey link within their professional networks.

#### Data analysis

Simple descriptive statistics such as frequency and percentages were used to quantitatively analyse and map the data. The types of studies, nature of projects, and aim of interventions were analysed using descriptive thematic analysis to map with predefined categories according to Meiland et al. (2017). The aims of projects in relation to the technology use were mapped to the following three main categories: 1-Devices intended to help manage their everyday life of people with dementia across the disease journey, 2-Technologies to help engage in meaningful and pleasurable activities, and 3-Health care technologies that aim to support professional organisations and systems (Meiland et al., 2017). The mapping was performed independently by two researchers (DS and LO) and conflicts were resolved through discussion and consensus.

#### Ethics

A research ethics committee approval was obtained from… was obtained from the University of Galway Research Ethics Committee with reference number 2021.11.015. The European Parliament and Council of the European Union (2016) were followed in order to protect participants’ data and privacy. All participants were asked to provide informed consent prior to answering the survey questions.

### Phase 2: Delphi consensus study

The second phase of this research employed a survey-based consensus method to synthesise recommendations on the use and development of AT for PABD. We used the Delphi method (Dalkey and Helmer, 1963), a well-established technique for achieving consensus among a group of experts (Rowe et al., 1991). The Delphi technique applies a feedback process that consists of a series of structured questionnaire rounds (Diamond et al., 2014; Powell, 2003). In this study, a 2-round consensus procedure was employed in line with the three principles of the Delphi technique: (a) anonymity during rating; (b) multiple rounds (minimum 2); and (c) feedback to respondents to inform them about each previous rating before they start the next round (Fink-Hafner et al., 2019; James and Warren-Forward, 2015).

#### Recruitment and respondents

The project had a steering group (current authors) which was composed of INTERDEM members and two members of Alzheimer Europe’s European Working Group of People with Dementia (EWGPWD). The steering group emphasised the importance of having a Delphi panel with diverse expertise in health and social care, policy, professional, and lived experiences (Gattrell et al., 2024). To achieve this, we reached out to all members of the INTERDEM organisation, members of EWGPWD and Alzheimer Europe’s European Dementia Carers Working Group (EDCWG). This approach ensured participation from various geographical locations, involving individuals from Ireland, the UK, Germany, the Netherlands, Italy, Portugal, and Sweden. While there is no universally agreed-upon ideal size for a Delphi panel (Akins et al., 2005), our panel size [*n* = 67 (round 1); *n* = 80 (round 2)] aligns with those in existing literature, ensuring sufficient responses despite withdrawals and partial completions. The recruitment of ‘experts by experience’ has been encouraged in this approach (Hardy et al., 2004) and serves to provide important primary contributions from the affected cohort.

#### Materials

During the rounds of the Delphi method, respondents completed online questionnaires via ‘Qualtrics’. They received a plain language statement (containing information about the taskforce and present study) and consent form at the outset, as well as debriefing materials (mentioning participants’ right to withdraw and providing contact details) at the end of the questionnaire. For each recommendation, respondents were asked to provide a rating from 1–10 regarding its *clarity* (1 = very difficult to understand, 10 = very easy to understand), *ease of implementation* (1 = very difficult to implement, 10 = very easy to implement), and *importance* (1 = not important, 10 = essential). They were also given the option to provide written feedback for each recommendation.

#### Statement generation

The recommendation statements were developed through an integrative process combining the findings of the mapping exercise (Phase 1) with those of Barbosa et al.’s (2023) scoping review. The results of the mapping exercise were first shared with the wider research team for review, after which an initial set of recommendations was independently derived from both the mapping and the literature review. These two sources were then systematically compared and synthesised into a consolidated list of statements, forming the basis for the first round of the Delphi process. In instances where overlapping content emerged, recommendations were merged to avoid redundancy. For example, the two recommendations of (a)*“Consider the use of robot pets may be an effective intervention for people with dementia who are experiencing loneliness or anxiety”* and (b)*“Consider the use of robot pets to increase social interaction for people with dementia”* were combined into the recommendation (c)*“The use of robot pets may be an effective intervention to increase social interaction in people with dementia who are experiencing loneliness or anxiety.”* The completion of this phase resulted in 28 recommendations, which we grouped thematically under the five topics: “Development of Technology”, “Policy”, “Technology in Practice”, “Supporting the Use of Technology”, and “Research”.

Two members of the task force who have dementia (from the EWGPWD), as well as AD and DG, reviewed this set of recommendations to ensure that the language used was appropriate and easy to understand. After receiving their feedback, minor amendments were made to the phraseology, and certain items were pruned from the overall set. After this process, the total set of recommendations was reduced to 20 items. This set of recommendations can be found in Appendix A.

#### Consensus round 1

In the first round of the Delphi process, the questionnaire was sent to all member of INTERDEM and the INTERDEM academy; current and past members of the EWGPWD with their primary carer. The questionnaire included 20 statements and respondents were asked to rate the clarity, ease of implementation, and importance of each statement. It was also possible to provide written feedback. The people with dementia and carers involved in this round included members of Alzheimer Europe’s EWGPWD – this constituted the ‘public involvement’ activities of this work. In the case of the members of the EWGPWD, the recommendations were discussed at a meeting facilitated by Alzheimer Europe and their combined feedback was included in a single document. All the feedback was included in preparation for Round 2.

#### Consensus round 2

The data collected in Round 1 was reviewed for each recommendation that did not achieve consensus. We made the appropriate changes to these statements, addressing the issues that were highlighted both in the quantitative (clarity, ease of implementation, and importance scores) and qualitative data (written comments). The phraseology was amended for 10 out of the 11 recommendations; we did not alter the wording of the recommendation ‘SUT3’ (see Appendix A), because the feedback suggested that the primary issues of concern with this recommendation centred around ease of implementation, with no concerns being raised around clarity/phraseology. A comparison of the recommendations between Rounds 1 and 2 can be found in the Appendix B. Further details regarding the changes made to the recommendations between the two consensus rounds are included in Supplementary File 1.

Following this, a Qualtrics-based survey was disseminated to the wider INTERDEM group which included the 11 recommendations that did not reach consensus, as well as the alterations that were made in order to address the issues that were highlighted. Respondents were asked to provide quantitative and qualitative feedback for the *amended* versions of these recommendations (with the exception of SUT3, for the reasons outlined above). Respondents only rated the aspects that had not reached consensus in the previous round. For example, if a statement reached consensus regarding importance – but not clarity – in Round 1, they only rated its clarity in Round 2. This approach ensured efficient and focused feedback on only the unresolved issues. Please note that, during the data collection in Round 2, we did not require respondents to evaluate the ‘ease of implementation’ of each recommendation. Our primary focus was to understand experts’ perceptions of the importance and clarity of these recommendations, rather than the technical feasibility of any given statement. Furthermore, the qualitative feedback from Round 1 did not suggest any specific phraseology adjustments—and it is likely that changes in wording here would not improve the ease of implementation scores for these recommendations. Therefore, in Round 2 respondents provided clarity and/or importance ratings for each recommendation.

#### Consensus criteria

Consensus was measured by assessing both the central tendency and dispersion of respondents’ ratings. Central tendency was measured by the median score on the 10-point Likert scale; a statement was considered to reach a *positive* consensus for this criterium (clear/important/easy to implement) if the median score was 8–10; *undecided* if the median was 4–7; and a *negative* consensus (unclear/unimportant/difficult to implement) if it was 1–3.

Dispersion was measured, firstly, by the quartile deviation (QD; measured by halving the interquartile range). A QD of ≤1 indicated a high consensus level for this criterium; 1 < QD < 2 indicated a moderate level of consensus; and QD > 2 indicated a low consensus level. These criteria were taken from Ab Latif et al. (2017) and adjusted for a 10-point Likert scale. Secondly, we considered the percentage of respondent responses that fell within the interquartile range (between Q1 and Q3). If this score was 70% or higher, the recommendation was considered to have reached consensus for this criterion. Furthermore, in Round 2, there was an additional criterion introduced; it was required that 51% of participants’ responses for any given recommendation must fall within the positive consensus range (8–10). Further details on the process by which overall consensus was determined are presented in Supplementary File 2.

#### Ethics

Ethical approval was not required for this aspect of the study. Respondents (experts in the field) provided their opinion on the recommendations. European Parliament and Council of the European Union (2016) were followed in order to protect respondents’ opinions and privacy.

## Results

### Phase 1: mapping exercise

In total, 48 responses were received and 31 were fully completed. All available responses were included in the analysis to gather all relevant information. A total of 28 projects were reported, almost all of which were submitted by participants from Europe (*n* = 26) with the majority reporting from the United Kingdom (*n* = 12). Seven projects were run or implemented by multiple countries. The data reported for 28 projects were categorised and mapped according to the framework by Meiland et al. (2017). The summary of the projects identified by the mapping exercise is included in Appendix C. The majority of the projects (*n* = 20) were mapped under the “Technologies to help engage in meaningful and pleasurable activities” category. Examples of the projects were musical activities facilitated using digital technologies, a mobile application for reminiscence therapy, the use of video calls for peer support, and digital game-based activities. Overall, video-conferencing platforms were widely used in these projects. Moreover, 17 of the projects or initiatives were adapted during the pandemic to utilise digital technology due to the restrictions during this time.

### Phase 2: Delphi consensus study

#### Consensus round 1

There were 67 respondents to the Round 1 survey, which was recorded between November 2022 and February 2023. There were 24 cases which were found to have missing data; these were removed, resulting in a total of 43 respondents. The sample consisted of members of the INTERDEM AT task force, and the wider INTERDEM group. In total, nine recommendations reached a high level of consensus at the end of the first round; therefore, the 11 recommendations that did not achieve a consensus advanced to the second round of the process. See Table 1 for details.

**Table 1:** Results for round 1 of the consensus process.

Recommendation	High consensus achieved	Clarity		Ease of implementation		Importance	
		Median	QD	Median	QD	Median	QD
Development of technology							
DOT1		8	1	6	1	10	1
DOT2		8	2	6	1	9	1
DOT3		6	2	6	1	8	1.5
DOT4	✓	9	1	7	1.5	10	1
Policy							
P1	✓	9	1	4	1	8	1.5
P2		9	1	5	1	8	1.5
Technology in practice							
TIP1	✓	8	2.5	6	1.5	8	1.5
TIP2	✓	9	1	8	1	8	1.5
TIP3		8	1	6	1.5	8	1.5
TIP4		8	1.5	6	1.5	7	1.5
TIP5	✓	9	1.5	8	1.5	10	1
TIP6		7	1.5	6	1.5	8	1
TIP7	✓	9	1.5	7	1	8	1.5
Supporting the use of technology							
SUT1		8	1.5	6	1.5	9	1
SUT2	✓	9	1	6	1.5	8	1.5
SUT3		9	1	5	2	8	1
SUT4		8	2	6	1.5	8	1.5
SUT5	✓	9	1	6	1	8	1.5
Research							
R1	✓	10	0.5	7	1	10	1
R2		9	1.5	7	1.5	8	1

QD, quartile deviation.

#### Consensus round 2

There were 80 respondents to the Round 2 survey, which was recorded between April and June 2023. Overall, 15 cases were found to have missing data and were removed; resulting in a total of 65 respondents in Round 2. This sample comprised 52 INTERDEM group members (who completed the survey via Qualtrics) and 13 members of the EWGPWD composed of people with dementia, who completed the survey using pen and paper. Out of the 11 recommendations included in Round 2, seven achieved a high level of consensus. Out of the remaining four statements, two (DOT1 and TIP4) achieved a moderate level of consensus, while two (DOT3 and TIP3) exhibited a low level of consensus. Please see Table 2 for further details. Considering these statistics, we removed DOT3 and TIP3, as these recommendations exhibited low consensus ratings across the board and during both rounds. We amended the phraseology of DOT1 and TIP4 to reflect the suggestions included in the respondents’ qualitative feedback from Round 2.

**Table 2:** Results for round 2 of the consensus process.

Recommendation	Consensus level achieved	Clarity				Importance			
		Median	QD	% in Q1-Q3	% in 8–10	Median	QD	% in Q1–Q3	% in 8–10
DOT1	Moderate	8	1.25	62.3	70.8				
DOT2	High	9	1.5	86.1	73.8				
DOT3	Low	6	2	56.9	33.8				
P2	High	9	1	79.9	80				
TIP3	Low	8	2	64.6	53.8				
TIP4	Moderate	8	1.25	60.1	56.9	8	1.5	67.7	55.4
TIP6	High					9	1	78.5	78.5
SUT1	High	8	1	58.4	63.1	8	0.75	64.6	76.9
SUT3	High					8	1.75	80.8	56.9
SUT4	High	9	1	80	80	9	1	78.5	78.5
R2	High					8	1.5	80.1	61.5

### Final recommendations

The consensus study resulted in 18 recommendations being synthesised. The final recommendations are listed in Table 3. Note that, for those items which are not followed by an “Original recommendation” paragraph in this Appendix (e.g., DOT4), consensus was achieved during Round 1. The term “supporters” was critiqued as too vague and replaced with ‘relatives/caregivers’ in items that reached Round 2 of the consensus process. Items TIP6, SUT2, SUT3, and SUT5, which reached consensus in Round 1 (and which included the term “supporters”), were therefore amended post hoc.

**Table 3:** The final set of synthesised recommendations.

Section	Code	Recommendation
Development of technology	DOT1	In order to increase the effectiveness and uptake of technology products, involve people with dementia and their relatives/caregivers during development.
	DOT2	Support from a relative or caregiver when setting up technology increases the likelihood of its success.
	DOT3	If the person with dementia is not familiar with the technology (e.g., if it is very new), provide support/training in how to use it if needed.
Policy	P1	Make digital inclusion a human right.
	P2	Provide free or subsidised access to technology to people with dementia, if they are unable to afford it themselves.
Technology in practice	TIP1	Offer both online and in-person opportunities to engage (e.g., GP consultations, outpatient clinics, Alzheimer Cafes, support groups to prevent social isolation).
	TIP2	Offer both video consultations (e.g., zoom) and telephone calls for remote consultations.
	TIP3	To overcome possible difficulties during online sessions, ensure that it is possible to control the technology remotely, if the person with dementia is comfortable with that.
	TIP4	When organising online activities, be attentive to time, the complexity of topics addressed, and the level of demands placed on people with dementia so as to avoid overloading them.
	TIP5	When using social robots, consider them an assistant tool rather than a replacement for real-life interaction.
	TIP6	Remote support groups/opportunities for reminiscence should be provided to people with dementia and their relatives/caregivers who are unable to attend in-person sessions.
Supporting the use of technology	SUT1	As not every person with dementia has a relative/caregiver, a healthcare provider should offer support to enable them to use the technology (e.g., video calls, robot pets).
	SUT2	Use specially-trained advisers to install technology and teach people with dementia and/or their relatives and caregivers how to use it.
	SUT3	Increase the availability of trained staff in hospitals, nursing homes and in the community to support people with dementia and their relatives/caregivers to use technology.
	SUT4	Increase awareness among care professionals of the barriers that some people may encounter when accessing telehealth services (e.g., cost, poor WiFi).
	SUT5	Ensure that different levels of support are available to people with dementia and their relatives/caregivers based on dementia severity and familiarity with technology.
Research	R1	Allocate funding to research into technology and dementia.
	R2	With regard to potential cross-cultural differences, do not assume that findings can be generalised.

## Discussion

The present study aimed to (a) conduct a mapping exercise to identify research studies and projects conducted during the COVID-19 pandemic that involved the use of digital technology by people with dementia, and (b) conduct a Delphi study, synthesising the results from this mapping exercise and the recommendations from Barbosa et al.’ (2023) scoping review for the development and use of assistive technology among people with dementia.

The mapping exercise identified 28 projects, of which 15 were initiated before the pandemic and the remaining 13 were commenced during the pandemic. Many projects were adapted to use digital technology during the pandemic and majority of them were categorised as “technologies to help engage in meaningful and pleasurable activities,” which could be for the purpose of replacing activities that had been usually provided in a face to face before the pandemic restrictions. The use of digital technology in different aspects of research studies and projects has several benefits to the researchers and participants such as not requiring travel time, allowing easy and resource-effective nationwide or international data collection, and maintaining the connection between the facilitator and the recipient at the time of national emergencies such as the pandemic restrictions. However, the implementation of (digital) technology in research studies or other projects involving people with dementia either as part of an intervention or a method of data collection requires careful planning to ensure an inclusive approach considering the expectations of digital literacy and familiarity/comfort with technology (Conway et al., 2023). Nonetheless, these projects delivered through digital technologies during the pandemic offered support to people with dementia and provided potential opportunities to improve their skills to enhance their digital literacy, which could further support their interactions and engagement in meaningful and pleasurable activities (Meiland et al., 2017; Talbot and Briggs, 2022). Another aspect of the mapping exercise was the identification of ‘public involvement’ use in the projects that utilised digital technology for people with dementia. An example of this was a project that investigated the use of everyday technology (e.g., over 90 everyday technologies including analogue and digital microwaves, GPS technologies and online banking) and its interplay in everyday life (Wallcook et al., 2020). Like many other projects, their ‘public involvement’ interactions were changed from face-to-face to a digital interface due to the pandemic. Considering the benefits of the technology, online communication platforms offered significant opportunities to continue the facilitation of ‘public involvement’ despite the challenges of the pandemic. The pandemic restrictions accelerated this transition to move to online public involvement, which is now the leading approach to co-production in dementia research (Molinari-Ulate et al., 2022).

Drawing on the mapping exercise and scoping review, the Delphi study produced a set of 18 evidence-based recommendations. These recommendations emphasise the importance of involving people with dementia and their relatives/caregivers in the development and implementation of technologies, ensuring equitable access, and providing adequate training and support to users. These measures are crucial for overcoming barriers to technology adoption, such as usability challenges, lack of digital literacy, and insufficient support structures. The mapping exercise and the Delphi study together provide critical insights into how technology adoption can be effectively supported and expanded for this population. Our findings echo the sentiments from previous research with, for example, a strong emphasis placed on the involvement of people with dementia in the conception, design, and development of AT, and stressing the importance of training (for installers and care staff), support, and including digital inclusion as a right in order to increase accessibility. Here, we also provide novel expert insights into the needs of people with dementia in this vein, such as calling for both online and in-person options for consultation; tailoring technological support based on each person’s familiarity with technology; and advocating for a culturally sensitive approach to research.

### Implications for policy and practice

This research culminates in recommendations for technology development, implementation, and use that can be understood under two tiers of action. Firstly, immediate, service-level steps can be enacted within existing workflows. We outline achievable goals, including the delivery of skills-based digital literacy programmes for people with dementia and relatives/caregivers; the provision of installation support via trained advisers; hybrid access to services (e.g., in-person/telephone/video consultations) with support according to symptom severity/technology familiarity; and highlighting non-medical barriers to telehealth to social-care workers (e.g., cost, connectivity). Integrating these feasible actions into existing frameworks can deliver tangible improvements in technology access and adoption among people with dementia.

Furthermore, this work highlights the need for more ambitious, structural changes, requiring policy-level initiatives. These include establishing the right to digital access; subsidising or providing devices/connectivity for those unable to afford them; and funding workforces to sustain ongoing technology support across homes, communities, and residential care. The recommendations highlight the need for European-level funding streams dedicated to digital inclusion, training programmes for dementia-capable digital care, and cross-sector collaborations that join health, social care, and telecoms to deliver equitable access. These directions align with the WHO Global Action Plan on Dementia (2017–2025), which emphasises using innovative health technologies, increasing the awareness of dementia in society, and collaborating with stakeholders in research. We add to this by addressing gaps around digital rights, minimum connectivity and device standards, and routine resourcing for hands-on support. Working across these scales and translating the current recommendations into actionable plans would enable the development of research-informed, rights-based practices that systematically support the well-being of people affected by dementia.

### Strengths and limitations

The merits of the current research should be highlighted. The combination of a mapping exercise and a Delphi consensus method allowed for a broad assessment of existing evidence and expert opinion, and the adherence to the ACCORD guideline ensures the comprehensive and standardised reporting of the present findings. Recommendations were derived from a broad and diverse body of literature, ensuring a representative range of perspectives. The inclusion of multidisciplinary experts, and ‘public involvement’, further enhanced the robustness and credibility of the findings, supporting a comprehensive understanding of the topic and mitigating potential biases. A rigorous consensus standard was maintained throughout the Delphi process, increasing the reliability of the resulting recommendations. In addition, qualitative analysis of written feedback strengthened the clarity and appropriateness of recommendation phrasing, as judged by experts with experience. Similarly, the mapping survey identified a broad range of examples of funded and voluntary research projects and other initiatives conducted during the pandemic to support people affected by dementia. The mapping survey data analysis followed a robust approach, guided by a framework widely used in dementia (Meiland et al., 2017).

Nonetheless, some limitations warrant acknowledgement. The mapping survey had a limited response rate and relied on self-reports. To address this, the survey was distributed through the professional networks of INTERDEM researchers, but this limited the ability to accurately determine the total sample size and response rate. While the sampling approach enabled us to capture projects from active dementia researchers and stakeholders across multiple countries, the convenience sampling methods mean the findings primarily reflect the views of researchers accessible through INTERDEM networks. We acknowledge that this limitation indicates a potential selection bias, and it restricts the generalisability of the findings. Despite the limitations, the mapping exercise identified and described numerous examples of technology use among people with dementia during the COVID-19 pandemic. Although the focus of the AT task force subgroup was primarily on assistive technology, the mapping exercise aimed to identify all relevant digital technologies. This broader scope was intentional, aiming to capture a wide range of technologies in use.

## Conclusion

This study provided valuable insights into current research endeavours concerning the role of technology in supporting people with dementia, particularly during the challenging circumstances of a global pandemic. The findings underscore the importance of involving end-users in the design and deployment of these technologies, ensuring accessibility, and providing robust support and training. The recommendations derived from this Delphi study offer a practical framework for supporting the use and development of AT in dementia care, with the potential to improve quality of life and promote greater independence for PABD. Importantly, these findings remain useful beyond the context of COVID-19 – the current findings offer guidance for integrating digital solutions into dementia care and fostering resilience, both in general daily living and in the face of future healthcare challenges. As outlined, these recommendations operate across immediate service-level actions and longer-term policy initiatives, providing a roadmap for actionable, practical, and structural progress in the use of technology in dementia care. Future research should continue to explore the long-term impacts of these technologies, and address the barriers identified, to maximise their benefits for this population.

## Data Availability

The datasets presented in this article are not readily available because this research contains information collected directly from researchers regarding their ongoing or completed projects. While some of these data may exist publicly in dispersed formats, the dataset itself was collated from individual contributors and is not otherwise publicly accessible in a consolidated form. Informed consent was provided for the current project only. Requests to access the datasets should be directed to DS, duygu.sezgin@universityofgalway.ie.
