# Supplementary material for: Understanding and guiding technology use in dementia: a pan-European mapping and consensus study
Source: Front Dement. 2025 Dec 17;4:1735879. doi: 10.3389/frdem.2025.1735879 (PMC12753415; doi:10.3389/frdem.2025.1735879)
Supplement: Supplementary file 1 [file Supplementary_file_1.docx]

***Section 1: Development of Technology***

**DOT1. ‘**In order to increase the effectiveness and uptake of technology products, involve people with dementia and their relatives/caregivers during development.’

***Original recommendation.* “**Involve people with dementia and/or their supporters in the development of technology products in order to increase effectiveness and uptake.” In the feedback following Round 1, the use of the word “supporters” was critiqued on account of its equivocacy and was therefore changed to “relatives/caregivers” - resulting in: “In order to increase the effectiveness and uptake of technology products, involve people with dementia and/or their relatives/caregivers during the development stage”. Following Round 2, the two primary arguments that emerged from the qualitative feedback seemed to voice concerns about focusing on the “development stage” (versus the development of technology products at all stages); as well as the use of “and/or”, therefore these issues were amended.

**DOT2.** ‘Support from a relative or caregiver when setting up technology increases the likelihood of its success.’

***Original recommendation.*** “Ensure the person with dementia has support, if needed, from a supporter when setting up technology as this increases the likelihood of its success”. In the feedback following Round 1, the use of the word “supporter” was again critiqued and was changed to “relative or caregiver”. This recommendation achieved consensus in Round 2.

**DOT4.** ‘If the person with dementia is not familiar with the technology (e.g. if it is very new), provide support/training in how to use it if needed.’

***Section 2: Policy***

**P1. ‘**Make digital inclusion a human right.’

**P2. ‘**Provide free or subsidised access to technology to people with dementia, if they are unable to afford it themselves.’

***Original recommendation.*** “Provide free or subsidised access to technology to people with dementia where necessary”. The qualitative feedback for Round 1 suggested that the term “where necessary” was redundant and unclear, and was therefore replaced with “if they are unable to afford it themselves”. This recommendation achieved consensus in Round 2.

***Section 3: Technology in Practice***

**TIP1. ‘**Offer both online and in-person opportunities to engage (e.g. GP consultations, outpatient clinics, Alzheimer Cafes, support groups to prevent social isolation).’

**TIP2. ‘**Offer both video consultations (e.g. zoom) and telephone calls for remote consultations.’

**TIP4. ‘**To overcome possible difficulties during online sessions, ensure that it is possible to control the technology remotely, if the person with dementia is comfortable with that.’

***Original recommendation.*** “Make sure that it is possible to control the technology remotely, if the person with dementia is comfortable with that, so as to help people overcome possible difficulties using the technology.” In the feedback following Round 1, this recommendation was critiqued for being verbose and relatively unclear and was therefore changed to: “To overcome possible difficulties during online consultations, ensure that it is possible to control the technology remotely, if the person with dementia is comfortable with that”. Following Round 2, feedback centred around the use of the term “online consultations”, which was generally thought to be too narrow, and was therefore changed to “online sessions” for the final recommendation.

**TIP5. ‘**When organising online activities, be attentive to time, the complexity of topics addressed and the level of demands placed on people with dementia so as to avoid overloading them.’

**TIP6. ‘**When using social robots, consider them an assistant tool rather than a replacement for real-life interaction.’

***Original recommendation.*** “In the interest of promoting human contact and social interaction with people with dementia, consider using robot pets, exergaming or similar technologies as possible supports but not as a replacement.” The qualitative feedback in Round 1 highlighted issues of verbosity and some pointed out that the purpose of the recommendation is unclear. The statement was rewritten with these issues in mind and achieved consensus in Round 2.

**TIP7. ‘**Remote support groups/opportunities for reminiscence should be provided to people with dementia and their supporters who are unable to attend in-person sessions.’

***Section 4: Supporting the Use of Technology***

**SUT1.** ‘As not every person with dementia has a relative/caregiver, a healthcare provider should offer support to enable them to use the technology (e.g., video calls, robot pets).’

***Original recommendation.*** “As not every person with dementia has a supporter, plan to provide support, if needed, to enable them to use the technology (e.g. video calls, robot pets)”. The qualitative feedback from Round 1 suggested it was unclear who should provide support, therefore we included “a healthcare provider should offer support…”. The use of the word “supporter” was also critiqued and, again,  replaced with “relative/caregiver”. This recommendation achieved consensus in Round 2.

**SUT2. ‘**Use specially-trained advisers to install technology and teach people with dementia and/or their supporters how to use it.’

**SUT3.** ‘Increase the availability of trained staff in hospitals, nursing homes and in the community to support people with dementia and their supporters to use technology.’

No amendment was made to the terminology or phraseology of this statement, as the concerns raised in the Round 1 feedback centred around the feasibility/ease of implementation of the recommendation, and not its wording/structure. The recommendation achieved consensus in Round 2.

**SUT4. ‘**Increase awareness among care professionals of the barriers that some people may encounter when accessing telehealth services (e.g., cost, poor WiFi).’

***Original recommendation.*** “Increase awareness among care professionals of the non-medical barriers to telehealth access”. The predominant request that emerged in Round 1’s qualitative feedback was to provide examples of “non-medical barriers”, therefore we included “e.g., cost, poor WiFi)”. There were also concerns voiced about phraseology. This recommendation achieved consensus in Round 2.

**SUT5. ‘**Ensure that different levels of support are available to people with dementia and their supporters based on dementia severity and familiarity with technology.’

***Section 5: Research***

**R1. ‘**Allocate funding to research into technology and dementia.’

**R2.** With regard to potential cross-cultural differences, do not assume that findings can be generalised.

***Original recommendation.*** “With regard to potential cross-cultural differences, do not assume that findings can be generalised across ‘Europe’”. The qualitative feedback from Round 1 strongly emphasised that the recommendation should acknowledge that research findings cannot be generalised across countries (or even within countries) due to cultural variation; therefore, we removed “across Europe”. The recommendation achieved consensus in Round 2.
