# Supplementary material for: Understanding and guiding technology use in dementia: a pan-European mapping and consensus study
Source: Front Dement. 2025 Dec 17;4:1735879. doi: 10.3389/frdem.2025.1735879 (PMC12753415; doi:10.3389/frdem.2025.1735879)
Supplement: Supplementary file 2 [file Supplementary_file_2.docx]

**Determining Overall Consensus.** We sought to uphold a rigorous and conservative standard of consensus by considering all of the aforementioned criteria in combination with participants’ written comments. In Round 1, a statement was considered to achieve an overall high level of consensus only if (a) the recommendation fulfilled the high-consensus criteria in at least two dimensions (i.e., clarity, ease of implementation, importance) for each of the dispersion and central tendency categories (i.e., median; QD; percentage of scores between Q1-Q3). In other words, to reach a high consensus, a statement necessarily exhibited a median of 8-10 (in at least two of the clarity, ease of implementation, and importance dimensions), a QD≤1 (again, in two out of these three dimensions), etc. Moreover, (b) a high consensus was achieved only if there were no significant and specific points of concern that arose consistently in the qualitative feedback. In Round 1, only statements which reached these criteria were considered to have achieved sufficient agreement to be excluded from the consensus process in the subsequent round.

In Round 2, we included an additional, intermediate status; a ‘moderate level of consensus’, which occurred if (a) between 50-62.5% of the high-level consensus criteria (i.e., median of 8-10; QD≤1; 70% of responses between Q1-Q3; *and* 51% of scores being 8-10) were fulfilled and (b) those criteria which did not reach a high level of consensus were judged to be approaching consensus. Please note again that, for this round, participants provided scores in terms of clarity *and/or* importance and, therefore, there were either four or eight consensus criteria per recommendation in this round. For example, consider the hypothetical Statement Z’s ‘Clarity’ ratings: Median = 8 [indicating high consensus]; QD = 1.25 [approaching high]; percentage of scores between Q1-Q3 = 60 [approaching high]; percentage of ratings between 8-10 = 70.8 [high consensus]. Here, 50% of the clarity ratings achieved a high level of agreement, while the remaining 50% were approaching consensus, indicating they were close to meeting the high-consensus criteria, and would therefore be considered as reaching a moderate level of consensus.
