# Supplementary material for: Understanding and guiding technology use in dementia: a pan-European mapping and consensus study
Source: Front Dement. 2025 Dec 17;4:1735879. doi: 10.3389/frdem.2025.1735879 (PMC12753415; doi:10.3389/frdem.2025.1735879)
Supplement: Supplementary file 3 [file Data_Sheet_1.docx]

**Appendix A**

**List of Recommendations Sent out in Round 1**

|  |  | **Development of Technology** |
| --- | --- | --- |
| 1 | DOT1 | Involve people with dementia and/or their supporters in the development of technology products in order to increase effectiveness and uptake. |
| 2 | DOT2 | Ensure the person with dementia has support, if needed, from a supporter when setting up technology as this increases the likelihood of its success. |
| 3 | DOT3 | Use the most straightforward, widely-available, video-communication software and launch with a single user action. |
| 4 | DOT4 | If the person with dementia is not familiar with the technology (e.g. if it is very new), provide support/training in how to use it if needed. |
|  |  | **Policy** |
| 5 | P1 | Make digital inclusion a human right |
| 6 | P2 | Provide free or subsidised access to technology to people with dementia where necessary. |
|  |  | **Technology in Practice** |
| 7 | TIP1 | Offer both online and in-person opportunities to engage (e.g. GP consultations, outpatient clinics, Alzheimer Cafes, support groups to prevent social isolation). |
| 8 | TIP2 | Offer both video consultations (e.g. zoom) and telephone calls for remote consultations. |
| 9 | TIP3 | Personalise online non-pharmacological interventions to make them more appealing. |
| 10 | TIP4 | Make sure that it is possible to control the technology remotely, if the person with dementia is comfortable with that, so as to help people overcome possible difficulties using the technology. |
| 11 | TIP5 | When organising online activities, be attentive to time, the complexity of topics addressed and the level of demands placed on people with dementia so as to avoid overloading them. |
| 12 | TIP6 | In the interest of promoting human contact and social interaction with people with dementia, consider using robot pets, exergaming or similar technologies as possible supports but not as a replacement. |
| 13 | TIP7 | Remote support groups/opportunities for reminiscence should be provided to people with dementia and their supporters who are unable to attend in-person sessions. |
|  |  | **Supporting the use of Technology** |
| 14 | SUT1 | As not every person with dementia has a supporter, plan to provide support, if needed, to enable them to use the technology (e.g. video calls, robot pets). |
| 15 | SUT2 | Use specially-trained advisers to install technology and teach people with dementia and/or their supporters how to use it |
| 16 | SUT3 | Increase the availability of trained staff in Hospitals, Nursing Homes and in the Community to support people with dementia and their supporters to use technology |
| 17 | SUT4 | Increase awareness among care professionals of the non-medical barriers to telehealth access |
| 18 | SUT5 | Ensure that different levels of support are available to people with dementia and their supporters based on dementia severity and familiarity with technology |
|  |  | **Research** |
| 19 | R1 | Allocate funding to research into technology and dementia. |
| 20 | R2 | With regard to potential cross-cultural differences, do not assume that findings can be generalised across "Europe". |

Appendix B

Changes made to Recommendations in Round 2

|  | Round 1 Version |  | Round 2 (Amended) Version |
| --- | --- | --- | --- |
| DOT1 | Involve people with dementia and/or their supporters in the development of technology products in order to increase effectiveness and uptake. |  | In order to increase the effectiveness and uptake of technology products, involve people with dementia and/or their relatives/caregivers during the development stage. |
| DOT2 | Ensure the person with dementia has support, if needed, from a supporter when setting up technology as this increases the likelihood of its success. |  | Support from a relative or caregiver when setting up technology increases the likelihood of its success. |
| DOT3 | Use the most straightforward, widely-available, video-communication software and launch with a single user action. |  | Use the most straightforward, widely-available, and secure video-communication software that can be launched without exiting the app. |
|  |  |  |  |
| P2 | Provide free or subsidised access to technology to people with dementia where necessary. |  | Provide free or subsidised access to technology to people with dementia, if they are unable to afford it themselves. |
|  |  |  |  |
| TIP3 | Personalise online non-pharmacological interventions to make them more appealing. |  | Non-pharmacological interventions should be tailored to the person as this makes them more appealing than generic interventions. |
| TIP4 | Make sure that it is possible to control the technology remotely, if the person with dementia is comfortable with that, so as to help people overcome possible difficulties using the technology. |  | To overcome possible difficulties during online consultations, ensure that it is possible to control the technology remotely, if the person with dementia is comfortable with that. |
| TIP6 | In the interest of promoting human contact and social interaction with people with dementia, consider using robot pets, exergaming or similar technologies as possible supports but not as a replacement. |  | When using social robots, consider them an assistant tool rather than a replacement for real-life interaction. |
|  |  |  |  |
| SUT1 | As not every person with dementia has a supporter, plan to provide support, if needed, to enable them to use the technology (e.g. video calls, robot pets). |  | As not every person with dementia has a relative/caregiver, a healthcare provider should offer support to enable them to use the technology (e.g., video calls, robot pets). |
| SUT3 | Increase the availability of trained staff in Hospitals, Nursing Homes and in the Community to support people with dementia and their supporters to use technology. |  | [No amendment was made to this statement as the issues raised centred around the feasibility/ease of implementation of the recommendation and not its wording/structure] |
| SUT4 | Increase awareness among care professionals of the non-medical barriers to telehealth access. |  | Increase awareness among care professionals of the barriers that some people may encounter when accessing telehealth services (e.g., cost, poor WiFi). |
|  |  |  |  |
| R2 | With regard to potential cross-cultural differences, do not assume that findings can be generalised across "Europe". |  | With regard to potential cross-cultural differences, do not assume that findings can be generalised. |

**Appendix C**

**UTeC19 Project: Mapping exercise, preliminary overview of results, June 2022**

|  | **Name of initiative/project/ activity** | **Country** | **Project commenced pre or during the pandemic?** | **Changed to a digital technology- based  project because of the pandemic?** | **Aim with technology** (Categories according to Meiland et al. 2017*) |
| --- | --- | --- | --- | --- | --- |
| 1 | Culture Box | United Kingdom | During | No | 2 |
| 2 | Music and technology against COVID-19: a case in older people | Spain | During | No | 2 |
| 3 | The InspireD Reminiscence Project | United Kingdom | Pre | Yes | 2 |
| 4 | Using videoconferencing to facilitate health and social care delivery during Covid-19: an interview study of care home staff. | United Kingdom | During | No | 3 |
| 5 | Mood-sense | The Netherlands | Pre | No | 3 |
| 6 | Peer support through video meetings for people with Young Onset Dementia | United Kingdom | Pre | No | 2 |
| 7 | Vichie/Virtually connected habitat for intergenerational activities/virtual room inspired by cave rooms for intergenerational activities involving people with dementia and preschool children | Italy | Pre | No | 2 |
| 8 | Puzzling the Mind | Switzerland | Pre | Yes | 2 |
| 9 | Virtual Individual Cognitive Stimulation Therapy in Hong Kong: a mixed methods feasibility study | Hong Kong | During | No | 2 |
| 10 | Virtual Individual Cognitive Stimulation Therapy: a feasibility randomised controlled trial | Hong Kong | During | No | 2 |
| 11 | Implementing Digital Singing in Dementia (IDISIDE) study | United Kingdom | During | Yes | 2 |
| 12 | CST-International | United Kingdom | During | Yes | 2 |
| 13 | Virtual CST – A collaborative proof of concept study with FaceCog HK in response to the Covid-19 pandemic | United Kingdom | During | No | 2 |
| 14 | Use of video consultations for families with dementia | United Kingdom | Pre | No | 3 |
| 15 | Digitale Aktivitetshold (Digital activity Communities) | Denmark | During | Yes | 2 |
|  |  |  |  |  |  |
|  | **Name of initiative/project/ activity, Contd.** | **Country** | **Project commenced pre or during the pandemic?** | **Changed to a digital technology- based  project because of the pandemic?** | **Aim with technology** (Categories according to Meiland et al. 2017*) |
| 16 | Trent Dementia empowerment programme | United Kingdom | Pre | Yes | 2 |
| 17 | Tea, Chats and Tunes | Ireland | Pre | Yes | 2 |
| 18 | Developing life stories through digital technology for persons with an intellectual disability and dementia. | Ireland | Pre | No | 2 |
| 19 | Cognisance (Co-designing dementia diagnosis and post diagnostic support) | The Netherlands | During | Yes | 1 |
| 20 | Communication with family | Czechia | Pre | Yes | 2 |
| 21 | Ordinary Care in Extraordinary times | Ireland | Pre | Yes |  |
| 22 | CAPTAIN - Coach Assistant via Projected and Tangible Interface And Groups of Experts by Experience (people with mild to moderate cognitive impairment, relatives and caregivers) | Spain | Pre | Yes | 1 2 3 |
| 23 | FindMyApps (a randomised controlled trial comparing the effect of the FindMyApps intervention with a normal ipad, with respect to social participation and self-management) | The Netherlands | Pre | Yes | 1 2 |
| 24 | Caban group a DEEP affiliated group of people living with dementia working with students and researchers using Zoom | United Kingdom | During | Yes | 1 2 |
| 25 | Supporting the management of long-term conditions in dementia | United Kingdom | During | Yes | 3 |
| 26 | Everyday Technology research and stakeholder involvement across the project including co-produced summary film of results | Sweden | Pre | Yes | 1 2 3 |
| 27 | Intergenerational activities | United Kingdom | Pre | Yes | 2 |
| 28 | COIL (impact of visitor ban in residential homes on the functioning of residents) | The Netherlands | During | Yes | 2  3 |

*Categorised as below, based on Meiland et al. 2017

**1**: Devices intended to help manage their everyday life across the disease journey

**2**: Technologies to help engage in meaningful and pleasurable activities

**3**: Health care technologies that aim to support professional organisations and systems
